# Should Out-Patients Pay?

**Published:** 1889-05-18

**Authors:** 


### Should Out-Patients Pay?

At Westminster Town Hall, on 30th ult., Dr. Gilbert Smith read a brief paper, with the above title, before the members of the Hospitals Association, Lord Carmarthen, M.P., in the chair. Dr. Gilbert Smith maintained that out-patients should not, under any circumstances, be made to pay; that it was impossible to use the honorary medical staff of the hospitals as consultants at provident dispensaries; that there was practically no abuse of the out-patient department, so far as patients seeking relief were concerned, and that any form of the pay system should be unreservedly condemned.

Mr. Timothy Holmes, F.R.C.S., opened the discussion by defending the provident dispensaries, and insisting upon the desirability of affiliating such institutions with the hospitals for out-patient work.

Dr. Bristowe, F.R.S., expressed general sympathy with the views of the two previous speakers, but desired a little more time before committing himself to a definite opinion for or against the proposition of the paper.

Mr. Nelson Hardy maintained the rights of the general practitioners, and declared that the poor-law dispensaries being adequately provided and managed, a majority of the persons now seeking relief as out-patients should be compelled to seek relief from these institutions. If this were done, very many of the present out-patients would consult private practitioners at their own expense, as they were well able to do.

Mr. Burdett, having pointed out that hospital treatment was intended, and must be secured, for the relief of the deserving poor above the pauper class, to the exclusion of well-to-do patients, vigorously defended the pay system. He, however, condemned the registration system, because in practice it produced more cruel results and injustice than any system yet associated with medical relief. He maintained that we were on a wrong tack altogether in England in the treatment of out-patients. Instead of endeavouring to ascertain the circumstances of every out-patient, or of a selected number of out-patients, by private enquiry, or through the Charity Organisation Society, the burden of proof should be thrown upon each patient who applied for relief. If a man went to a doctor he had to pay him his fee, and it was but just that if people went to a hospital out-patient department they should prove to the authorities, between the date of their first and subsequent visits, that they were fit objects to receive free medical relief. This system, combined with a voluntary assessment of such patients as would be willing to pay something per week during the time of their attendance at the hospital, might be seen at work at the Central London Ear and Throat Hospital, Gray's-inn-road. Anyone who would take the trouble to go to that institution, and compare the class of out-patients' work done there with that undertaken at the large general hospitals in London, would receive a revelation, as he could not fail to realise that the patients were drawn from the same classes, and the system in force in Gray's-inn-road could be equally well enforced throughout London, if the authorities chose to introduce it. The key to the solution of the out-patient problem was the abolition of free medical service at the hospitals. When every medical man attached to a hospital was paid for his services, as he ought to be paid, then the abuse of hospitals would disappear like morning mists before the rising sun.

Sir Sydney Waterlow closed the discussion by briefly stating that he considered the system in vogue at St. Bartholomew's Hospital the best in London, and in his opinion there was no abuse of any kind attaching to the out-patient system there. He took Mr. Nelson Hardy to task for wishing to make every out-patient a pauper—an iniquitous suggestion in his opinion, and one that would prove as dangerous in practice as it would be disgraceful to the age that adopted it.

Lord Carmarthen expressed the pleasure and profit he had derived from the discussion, declared his great interest in The Hospitals Association, and his desire, as chairman of its council, to promote its success to the fullest possible extent.

